# Theory, Methods, and Applications of Fractional Calculus

**DOI:** 10.1155/2014/249717

**Published:** 2014-06-09

**Authors:** Abdon Atangana, Adem Kiliçman, Suares Clovis Oukouomi Noutchie, Aydin Secer, Santanu Saha Ray, Ahmed M. A. El-Sayed

**Affiliations:** ^1^Institute for Groundwater Studies, University of the Free State, Bloemfontein 9300, South Africa; ^2^Department of Mathematics and Institute for Mathematical Research, Universiti Putra Malaysia, 43400 Serdang, Selangor, Malaysia; ^3^Department of Mathematical Sciences, North-West University, Mafikeng Campus, Mmabatho 2735, South Africa; ^4^Department of Mathematical Engineering, Yildiz Technical University, 34210 Istanbul, Turkey; ^5^Department of Mathematics, National Institute of Technology, Rourkela, Orissa 769 008, India; ^6^Mathematics Department, Faculty of Science, Alexandria University, Alexandria 21526, Egypt

Fractional calculus, in the understanding of its theoretical and real-world presentations in numerous regulations, for example, astronomy and manufacturing problems, is discovered to be accomplished of pronouncing phenomena owning long range memory special effects that are challenging to handle through traditional integer-order calculus. Nearby an increasing concentration has been in the modification of fractional calculus as a successful modelling instrument for complicated systems, contributing to innovative viewpoints in their dynamical investigation and regulator. This improvement in the methodical knowledge is established by an enormous quantity of evens developing on the subject, manuscripts, and presentations in the past years. Nevertheless, countless singularities still pose significant confronts to the apprehensive population and fractional calculus appears to be plausibly contestant to incorporate larger exemplars through detaching graceful dependent on the explanation of involvedness.

This special issue contains papers about recent theoretical development and methods and applications results on the topics in almost all branches of sciences and engineering. We have received 56 papers during the submission period. Five were withdrawn; 34 were rejected including the papers submitted to the member of our editorial board. Only 17 good papers were accepted for publication.

The papers of this special issue cover some new algorithms and procedures designed to explore conventional, fractional, and time-scales differential equations of general interest. New understandings of existences and uniqueness theorems of some differential equations were also offered. In the following we give the brief summary of the content of the special issue. The existence and uniqueness theorems for impulsive fractional equations with the two-point and integral boundary conditions and sufficient condition on the fractional integral for the convergence of a function were presented. Besides the stability, boundedness, and Lagrange stability of fractional differential equation with initial time difference, stability of nonlinear Dirichlet BVPs governed by fractional Laplacian was proposed. A novel study on the singular perturbations fractional equations, analysis of a fractional-order couple model with acceleration in feelings, *q*-Sumudu transforms of *q*-analogues of Bessel functions, certain fractional integral formulas involving the product of generalized Bessel functions, and an expansion formula with higher-order derivatives for fractional operators of variable order were investigated in detail. A novel study underpinning construction of solution for fractional differential equations such as decomposition method for time fractional reactional-diffusion equation and high-order compact difference scheme for numerical solution of time fractional heat equation, a procedure to construct exact solutions of nonlinear fractional differential equations, were presented. An investigation on impulsive multiterm fractional differential equations and multiple positive solutions for nonlinear fractional boundary value problems were undertaken.

The editorial board trust that the set of nominated papers will offer readers an opportune renovate of significant investigation subjects and may also operate as a policy for encouraging additional contribution in this fast evolving ground.

